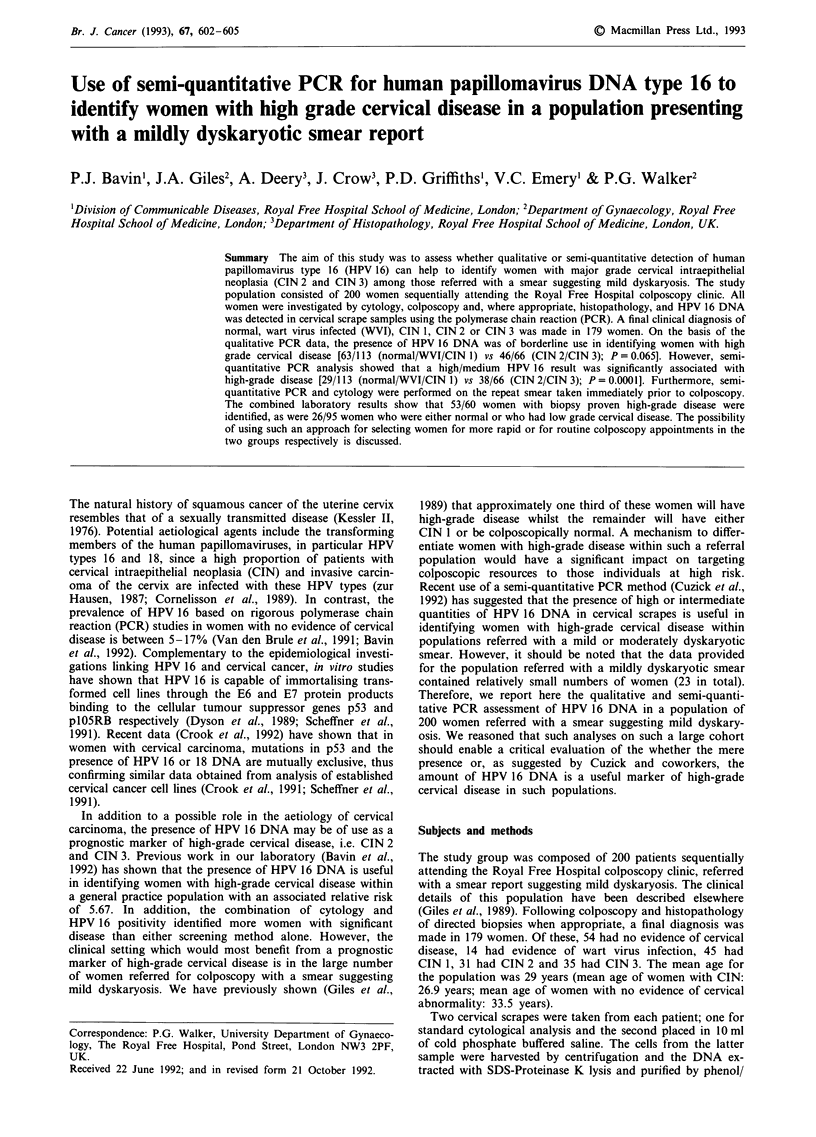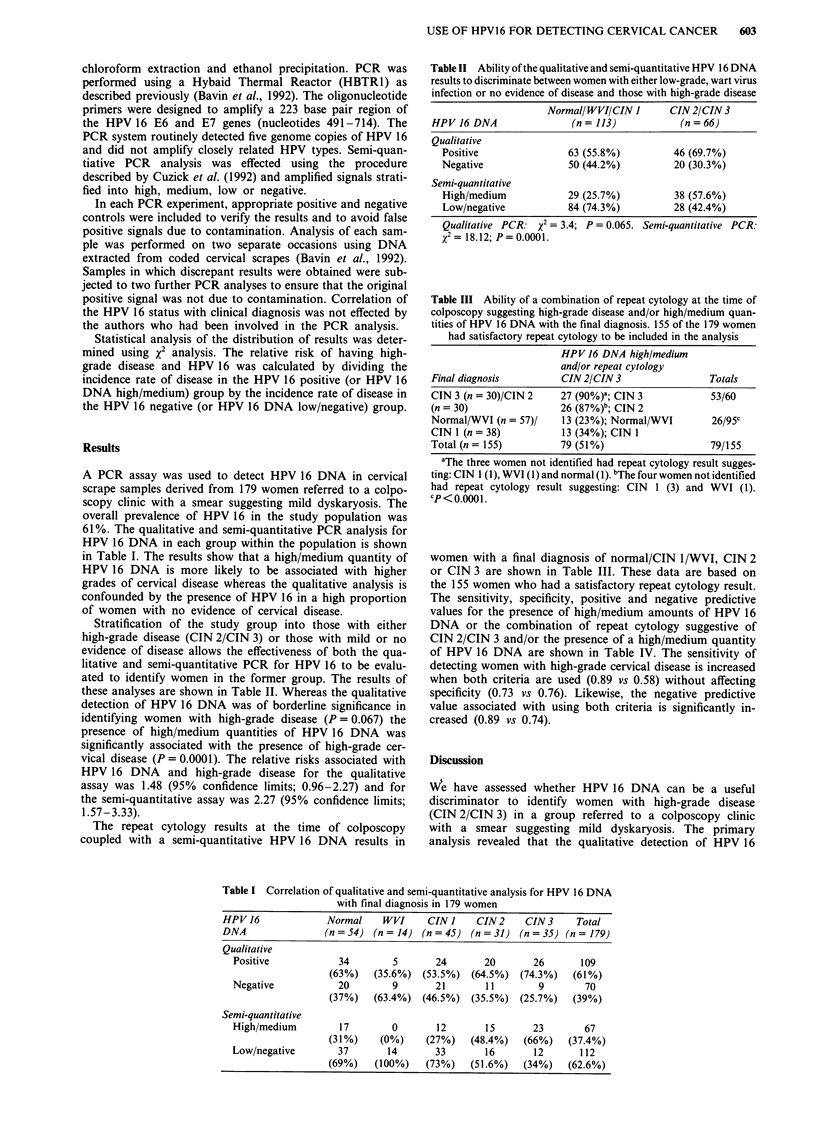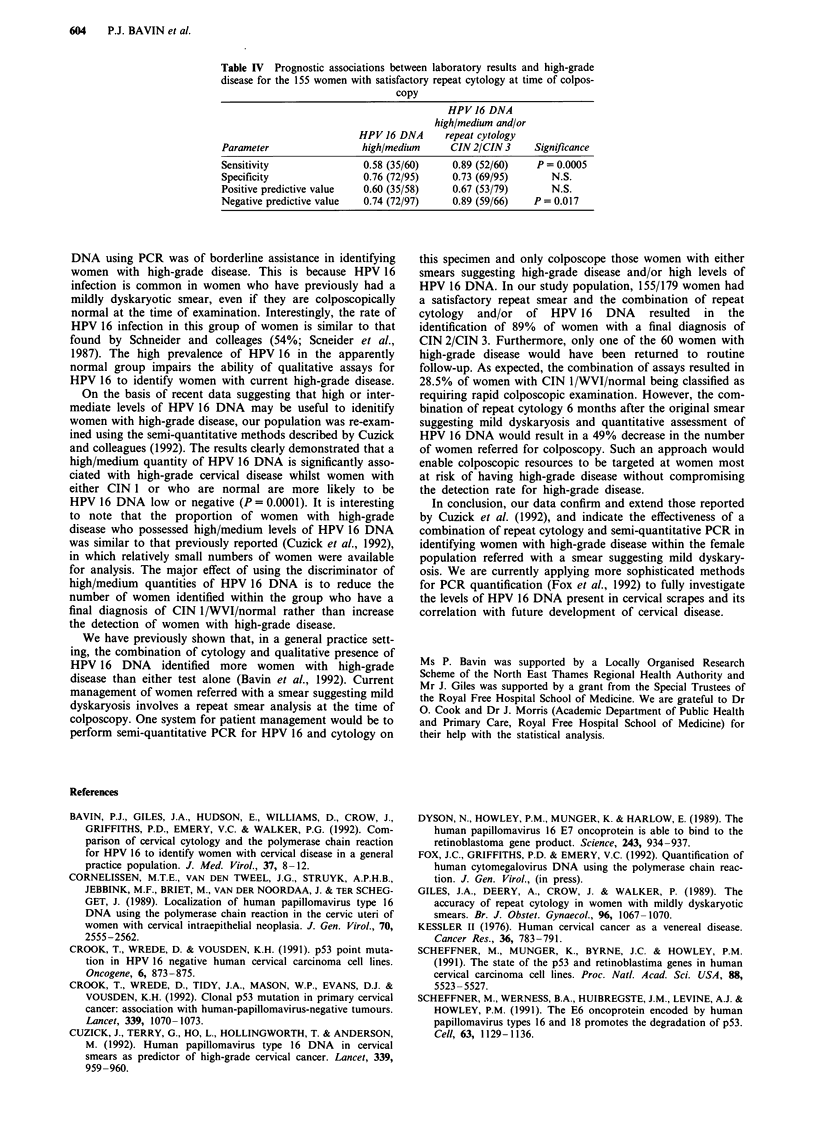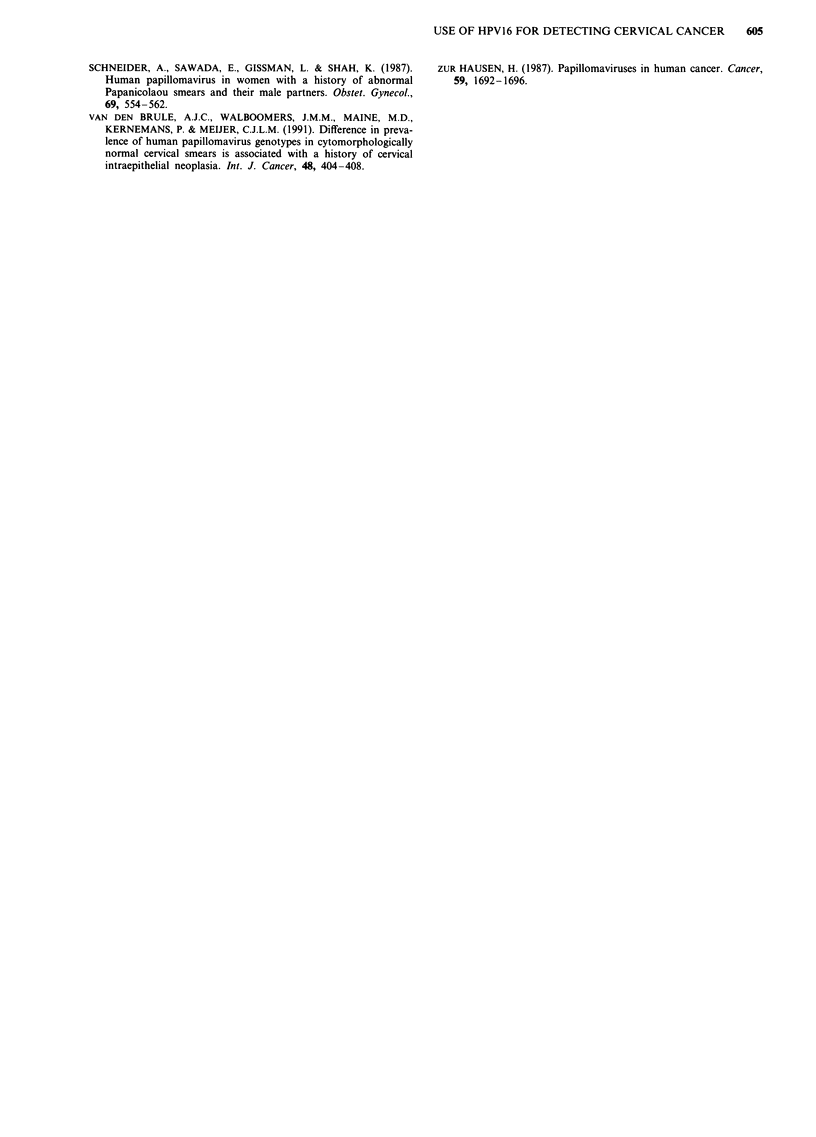# Use of semi-quantitative PCR for human papillomavirus DNA type 16 to identify women with high grade cervical disease in a population presenting with a mildly dyskaryotic smear report.

**DOI:** 10.1038/bjc.1993.110

**Published:** 1993-03

**Authors:** P. J. Bavin, J. A. Giles, A. Deery, J. Crow, P. D. Griffiths, V. C. Emery, P. G. Walker

**Affiliations:** Division of Communicable Diseases, Royal Free Hospital School of Medicine, London, UK.

## Abstract

The aim of this study was to assess whether qualitative or semi-quantitative detection of human papillomavirus type 16 (HPV 16) can help to identify women with major grade cervical intraepithelial neoplasia (CIN 2 and CIN 3) among those referred with a smear suggesting mild dyskaryosis. The study population consisted of 200 women sequentially attending the Royal Free Hospital colposcopy clinic. All women were investigated by cytology, colposcopy and, where appropriate, histopathology, and HPV 16 DNA was detected in cervical scrape samples using the polymerase chain reaction (PCR). A final clinical diagnosis of normal, wart virus infected (WVI), CIN 1, CIN 2 or CIN 3 was made in 179 women. On the basis of the qualitative PCR data, the presence of HPV 16 DNA was of borderline use in identifying women with high grade cervical disease [63/113 (normal/WVI/CIN 1) vs 46/66 (CIN 2/CIN 3); P = 0.065]. However, semi-quantitative PCR analysis showed that a high/medium HPV 16 result was significantly associated with high-grade disease [29/113 (normal/WVI/CIN 1) vs 38/66 (CIN 2/CIN 3); P = 0.0001]. Furthermore, semi-quantitative PCR and cytology were performed on the repeat smear taken immediately prior to colposcopy. The combined laboratory results show that 53/60 women with biopsy proven high-grade disease were identified, as were 26/95 women who were either normal or who had low grade cervical disease. The possibility of using such an approach for selecting women for more rapid or for routine colposcopy appointments in the two groups respectively is discussed.


Br. J. Cancer (1993), 67, 602–605 © Macmillan Press Ltd., 1993

### Use of semi-quantitative PCR for human papillomavirus DNA type 16 to identify women with high grade cervical disease in a population presenting with a mildly dyskaryotic smear report

P.J. Bavin[1], J.A. Giles[2], A. Deery[3], J. Crow[3], P.D. Griffiths[1], V.C. Emery[1] & P.G. Walker[2]

[1]*Division of Communicable Diseases, Royal Free Hospital School of Medicine, London;* [2]*Department of Gynaecology, Royal Free Hospital School of Medicine, London;* [3]*Department of Histopathology, Royal Free Hospital School of Medicine, London, UK.*

**Summary** The aim of this study was to assess whether qualitative or semi-quantitative detection of human papillomavirus type 16 (HPV 16) can help to identify women with major grade cervical intraepithelial neoplasia (CIN 2 and CIN 3) among those referred with a smear suggesting mild dyskaryosis. The study population consisted of 200 women sequentially attending the Royal Free Hospital colposcopy clinic. All women were investigated by cytology, colposcopy and, where appropriate, histopathology, and HPV 16 DNA was detected in cervical scrape samples using the polymerase chain reaction (PCR). A final clinical diagnosis of normal, wart virus infected (WVI), CIN 1, CIN 2 or CIN 3 was made in 179 women. On the basis of the qualitative PCR data, the presence of HPV 16 DNA was of borderline use in identifying women with high grade cervical disease [63/113 (normal/WVI/CIN 1) *vs* 46/66 (CIN 2/CIN 3); $P = 0.065$]. However, semi-quantitative PCR analysis showed that a high/medium HPV 16 result was significantly associated with high-grade disease [29/113 (normal/WVI/CIN 1) *vs* 38/66 (CIN 2/CIN 3); $P = 0.0001$]. Furthermore, semi-quantitative PCR and cytology were performed on the repeat smear taken immediately prior to colposcopy. The combined laboratory results show that 53/60 women with biopsy proven high-grade disease were identified, as were 26/95 women who were either normal or who had low grade cervical disease. The possibility of using such an approach for selecting women for more rapid or for routine colposcopy appointments in the two groups respectively is discussed.

The natural history of squamous cancer of the uterine cervix resembles that of a sexually transmitted disease (Kessler II, 1976). Potential aetiological agents include the transforming members of the human papillomaviruses, in particular HPV types 16 and 18, since a high proportion of patients with cervical intraepithelial neoplasia (CIN) and invasive carcinoma of the cervix are infected with these HPV types (zur Hausen, 1987; Cornelisson *et al.*, 1989). In contrast, the prevalence of HPV 16 based on rigorous polymerase chain reaction (PCR) studies in women with no evidence of cervical disease is between 5–17% (Van den Brule *et al.*, 1991; Bavin *et al.*, 1992). Complementary to the epidemiological investigations linking HPV 16 and cervical cancer, *in vitro* studies have shown that HPV 16 is capable of immortalising transformed cell lines through the E6 and E7 protein products binding to the cellular tumour suppressor genes p53 and p105RB respectively (Dyson *et al.*, 1989; Scheffner *et al.*, 1991). Recent data (Crook *et al.*, 1992) have shown that in women with cervical carcinoma, mutations in p53 and the presence of HPV 16 or 18 DNA are mutually exclusive, thus confirming similar data obtained from analysis of established cervical cancer cell lines (Crook *et al.*, 1991; Scheffner *et al.*, 1991).

In addition to a possible role in the aetiology of cervical carcinoma, the presence of HPV 16 DNA may be of use as a prognostic marker of high-grade cervical disease, i.e. CIN 2 and CIN 3. Previous work in our laboratory (Bavin *et al.*, 1992) has shown that the presence of HPV 16 DNA is useful in identifying women with high-grade cervical disease within a general practice population with an associated relative risk of 5.67. In addition, the combination of cytology and HPV 16 positivity identified more women with significant disease than either screening method alone. However, the clinical setting which would most benefit from a prognostic marker of high-grade cervical disease is in the large number of women referred for colposcopy with a smear suggesting mild dyskaryosis. We have previously shown (Giles *et al.*, 1989) that approximately one third of these women will have high-grade disease whilst the remainder will have either CIN 1 or be colposcopically normal. A mechanism to differentiate women with high-grade disease within such a referral population would have a significant impact on targeting colposcopic resources to those individuals at high risk. Recent use of a semi-quantitative PCR method (Cuzick *et al.*, 1992) has suggested that the presence of high or intermediate quantities of HPV 16 DNA in cervical scrapes is useful in identifying women with high-grade cervical disease within populations referred with a mild or moderately dyskaryotic smear. However, it should be noted that the data provided for the population referred with a mildly dyskaryotic smear contained relatively small numbers of women (23 in total). Therefore, we report here the qualitative and semi-quantitative PCR assessment of HPV 16 DNA in a population of 200 women referred with a smear suggesting mild dyskaryosis. We reasoned that such analyses on such a large cohort should enable a critical evaluation of the whether the mere presence or, as suggested by Cuzick and coworkers, the amount of HPV 16 DNA is a useful marker of high-grade cervical disease in such populations.

### Subjects and methods

The study group was composed of 200 patients sequentially attending the Royal Free Hospital colposcopy clinic, referred with a smear report suggesting mild dyskaryosis. The clinical details of this population have been described elsewhere (Giles *et al.*, 1989). Following colposcopy and histopathology of directed biopsies when appropriate, a final diagnosis was made in 179 women. Of these, 54 had no evidence of cervical disease, 14 had evidence of wart virus infection, 45 had CIN 1, 31 had CIN 2 and 35 had CIN 3. The mean age for the population was 29 years (mean age of women with CIN: 26.9 years; mean age of women with no evidence of cervical abnormality: 33.5 years).

Two cervical scrapes were taken from each patient; one for standard cytological analysis and the second placed in 10 ml of cold phosphate buffered saline. The cells from the latter sample were harvested by centrifugation and the DNA extracted with SDS-Proteinase K lysis and purified by phenol/

Correspondence: P.G. Walker, University Department of Gynaecology, The Royal Free Hospital, Pond Street, London NW3 2PF, UK.

Received 22 June 1992; and in revised form 21 October 1992.

chloroform extraction and ethanol precipitation. PCR was performed using a Hybaid Thermal Reactor (HBTR1) as described previously (Bavin *et al.*, 1992). The oligonucleotide primers were designed to amplify a 223 base pair region of the HPV 16 E6 and E7 genes (nucleotides 491–714). The PCR system routinely detected five genome copies of HPV 16 and did not amplify closely related HPV types. Semi-quantiative PCR analysis was effected using the procedure described by Cuzick *et al.* (1992) and amplified signals stratified into high, medium, low or negative.

In each PCR experiment, appropriate positive and negative controls were included to verify the results and to avoid false positive signals due to contamination. Analysis of each sample was performed on two separate occasions using DNA extracted from coded cervical scrapes (Bavin *et al.*, 1992). Samples in which discrepant results were obtained were subjected to two further PCR analyses to ensure that the original positive signal was not due to contamination. Correlation of the HPV 16 status with clinical diagnosis was not effected by the authors who had been involved in the PCR analysis.

Statistical analysis of the distribution of results was determined using $\chi^2$ analysis. The relative risk of having high-grade disease and HPV 16 was calculated by dividing the incidence rate of disease in the HPV 16 positive (or HPV 16 DNA high/medium) group by the incidence rate of disease in the HPV 16 negative (or HPV 16 DNA low/negative) group.

### Results

A PCR assay was used to detect HPV 16 DNA in cervical scrape samples derived from 179 women referred to a colposcopy clinic with a smear suggesting mild dyskaryosis. The overall prevalence of HPV 16 in the study population was 61%. The qualitative and semi-quantitative PCR analysis for HPV 16 DNA in each group within the population is shown in Table I. The results show that a high/medium quantity of HPV 16 DNA is more likely to be associated with higher grades of cervical disease whereas the qualitative analysis is confounded by the presence of HPV 16 in a high proportion of women with no evidence of cervical disease.

Stratification of the study group into those with either high-grade disease (CIN 2/CIN 3) or those with mild or no evidence of disease allows the effectiveness of both the qualitative and semi-quantitative PCR for HPV 16 to be evaluated to identify women in the former group. The results of these analyses are shown in Table II. Whereas the qualitative detection of HPV 16 DNA was of borderline significance in identifying women with high-grade disease ($P = 0.067$) the presence of high/medium quantities of HPV 16 DNA was significantly associated with the presence of high-grade cervical disease ($P = 0.0001$). The relative risks associated with HPV 16 DNA and high-grade disease for the qualitative assay was 1.48 (95% confidence limits; 0.96–2.27) and for the semi-quantitative assay was 2.27 (95% confidence limits; 1.57–3.33).

The repeat cytology results at the time of colposcopy coupled with a semi-quantitative HPV 16 DNA results in women with a final diagnosis of normal/CIN 1/WVI, CIN 2 or CIN 3 are shown in Table III. These data are based on the 155 women who had a satisfactory repeat cytology result. The sensitivity, specificity, positive and negative predictive values for the presence of high/medium amounts of HPV 16 DNA or the combination of repeat cytology suggestive of CIN 2/CIN 3 and/or the presence of a high/medium quantity of HPV 16 DNA are shown in Table IV. The sensitivity of detecting women with high-grade cervical disease is increased when both criteria are used (0.89 *vs* 0.58) without affecting specificity (0.73 *vs* 0.76). Likewise, the negative predictive value associated with using both criteria is significantly increased (0.89 *vs* 0.74).

**Table II** Ability of the qualitative and semi-quantitative HPV 16 DNA results to discriminate between women with either low-grade, wart virus infection or no evidence of disease and those with high-grade disease

| *HPV 16 DNA* | *Normal/WVI/CIN 1 (n = 113)* | *CIN 2/CIN 3 (n = 66)* |
|---|---|---|
| *Qualitative* | | |
| Positive | 63 (55.8%) | 46 (69.7%) |
| Negative | 50 (44.2%) | 20 (30.3%) |
| *Semi-quantitative* | | |
| High/medium | 29 (25.7%) | 38 (57.6%) |
| Low/negative | 84 (74.3%) | 28 (42.4%) |

*Qualitative PCR:* $\chi^2 = 3.4$; $P = 0.065$. *Semi-quantitative PCR:* $\chi^2 = 18.12$; $P = 0.0001$.

**Table III** Ability of a combination of repeat cytology at the time of colposcopy suggesting high-grade disease and/or high/medium quantities of HPV 16 DNA with the final diagnosis. 155 of the 179 women had satisfactory repeat cytology to be included in the analysis

| *Final diagnosis* | *HPV 16 DNA high/medium and/or repeat cytology CIN 2/CIN 3* | *Totals* |
|---|---|---|
| CIN 3 (*n* = 30)/CIN 2 | 27 (90%)[a]; CIN 3 | 53/60 |
| (*n* = 30) | 26 (87%)[b]; CIN 2 | |
| Normal/WVI (*n* = 57)/ | 13 (23%); Normal/WVI | 26/95[c] |
| CIN 1 (*n* = 38) | 13 (34%); CIN 1 | |
| Total (*n* = 155) | 79 (51%) | 79/155 |

[a]The three women not identified had repeat cytology result suggesting: CIN 1 (1), WVI (1) and normal (1). [b]The four women not identified had repeat cytology result suggesting: CIN 1 (3) and WVI (1). [c]$P < 0.0001$.

### Discussion

We have assessed whether HPV 16 DNA can be a useful discriminator to identify women with high-grade disease (CIN 2/CIN 3) in a group referred to a colposcopy clinic with a smear suggesting mild dyskaryosis. The primary analysis revealed that the qualitative detection of HPV 16

**Table I** Correlation of qualitative and semi-quantitative analysis for HPV 16 DNA with final diagnosis in 179 women

| *HPV 16 DNA* | *Normal (n = 54)* | *WVI (n = 14)* | *CIN 1 (n = 45)* | *CIN 2 (n = 31)* | *CIN 3 (n = 35)* | *Total (n = 179)* |
|---|---|---|---|---|---|---|
| *Qualitative* | | | | | | |
| Positive | 34 (63%) | 5 (35.6%) | 24 (53.5%) | 20 (64.5%) | 26 (74.3%) | 109 (61%) |
| Negative | 20 (37%) | 9 (63.4%) | 21 (46.5%) | 11 (35.5%) | 9 (25.7%) | 70 (39%) |
| *Semi-quantitative* | | | | | | |
| High/medium | 17 (31%) | 0 (0%) | 12 (27%) | 15 (48.4%) | 23 (66%) | 67 (37.4%) |
| Low/negative | 37 (69%) | 14 (100%) | 33 (73%) | 16 (51.6%) | 12 (34%) | 112 (62.6%) |

Table IV Prognostic associations between laboratory results and high-grade disease for the 155 women with satisfactory repeat cytology at time of colposcopy

| Parameter | HPV 16 DNA high/medium | HPV 16 DNA high/medium and/or repeat cytology CIN 2/CIN 3 | Significance |
|---|---|---|---|
| Sensitivity | 0.58 (35/60) | 0.89 (52/60) | $P = 0.0005$ |
| Specificity | 0.76 (72/95) | 0.73 (69/95) | N.S. |
| Positive predictive value | 0.60 (35/58) | 0.67 (53/79) | N.S. |
| Negative predictive value | 0.74 (72/97) | 0.89 (59/66) | $P = 0.017$ |

DNA using PCR was of borderline assistance in identifying women with high-grade disease. This is because HPV 16 infection is common in women who have previously had a mildly dyskaryotic smear, even if they are colposcopically normal at the time of examination. Interestingly, the rate of HPV 16 infection in this group of women is similar to that found by Schneider and colleagues (54%; Scneider *et al.*, 1987). The high prevalence of HPV 16 in the apparently normal group impairs the ability of qualitative assays for HPV 16 to identify women with current high-grade disease.

On the basis of recent data suggesting that high or intermediate levels of HPV 16 DNA may be useful to idenitify women with high-grade disease, our population was re-examined using the semi-quantitative methods described by Cuzick and colleagues (1992). The results clearly demonstrated that a high/medium quantity of HPV 16 DNA is significantly associated with high-grade cervical disease whilst women with either CIN 1 or who are normal are more likely to be HPV 16 DNA low or negative ($P = 0.0001$). It is interesting to note that the proportion of women with high-grade disease who possessed high/medium levels of HPV 16 DNA was similar to that previously reported (Cuzick *et al.*, 1992), in which relatively small numbers of women were available for analysis. The major effect of using the discriminator of high/medium quantities of HPV 16 DNA is to reduce the number of women identified within the group who have a final diagnosis of CIN 1/WVI/normal rather than increase the detection of women with high-grade disease.

We have previously shown that, in a general practice setting, the combination of cytology and qualitative presence of HPV 16 DNA identified more women with high-grade disease than either test alone (Bavin *et al.*, 1992). Current management of women referred with a smear suggesting mild dyskaryosis involves a repeat smear analysis at the time of colposcopy. One system for patient management would be to perform semi-quantitative PCR for HPV 16 and cytology on this specimen and only colposcope those women with either smears suggesting high-grade disease and/or high levels of HPV 16 DNA. In our study population, 155/179 women had a satisfactory repeat smear and the combination of repeat cytology and/or of HPV 16 DNA resulted in the identification of 89% of women with a final diagnosis of CIN 2/CIN 3. Furthermore, only one of the 60 women with high-grade disease would have been returned to routine follow-up. As expected, the combination of assays resulted in 28.5% of women with CIN 1/WVI/normal being classified as requiring rapid colposcopic examination. However, the combination of repeat cytology 6 months after the original smear suggesting mild dyskaryosis and quantitative assessment of HPV 16 DNA would result in a 49% decrease in the number of women referred for colposcopy. Such an approach would enable colposcopic resources to be targeted at women most at risk of having high-grade disease without compromising the detection rate for high-grade disease.

In conclusion, our data confirm and extend those reported by Cuzick *et al.* (1992), and indicate the effectiveness of a combination of repeat cytology and semi-quantitative PCR in identifying women with high-grade disease within the female population referred with a smear suggesting mild dyskaryosis. We are currently applying more sophisticated methods for PCR quantification (Fox *et al.*, 1992) to fully investigate the levels of HPV 16 DNA present in cervical scrapes and its correlation with future development of cervical disease.

Ms P. Bavin was supported by a Locally Organised Research Scheme of the North East Thames Regional Health Authority and Mr J. Giles was supported by a grant from the Special Trustees of the Royal Free Hospital School of Medicine. We are grateful to Dr O. Cook and Dr J. Morris (Academic Department of Public Health and Primary Care, Royal Free Hospital School of Medicine) for their help with the statistical analysis.

### References

BAVIN, P.J., GILES, J.A., HUDSON, E., WILLIAMS, D., CROW, J., GRIFFITHS, P.D., EMERY, V.C. & WALKER, P.G. (1992). Comparison of cervical cytology and the polymerase chain reaction for HPV 16 to identify women with cervical disease in a general practice population. *J. Med. Virol.*, **37**, 8-12.

CORNELISSEN, M.T.E., VAN DEN TWEEL, J.G., STRUYK, A.P.H.B., JEBBINK, M.F., BRIET, M., VAN DER NOORDAA, J. & TER SCHEGGET, J. (1989). Localization of human papillomavirus type 16 DNA using the polymerase chain reaction in the cervic uteri of women with cervical intraepithelial neoplasia. *J. Gen. Virol.*, **70**, 2555-2562.

CROOK, T., WREDE, D. & VOUSDEN, K.H. (1991). p53 point mutation in HPV 16 negative human cervical carcinoma cell lines. *Oncogene*, **6**, 873-875.

CROOK, T., WREDE, D., TIDY, J.A., MASON, W.P., EVANS, D.J. & VOUSDEN, K.H. (1992). Clonal p53 mutation in primary cervical cancer: association with human-papillomavirus-negative tumours. *Lancet*, **339**, 1070-1073.

CUZICK, J., TERRY, G., HO, L., HOLLINGWORTH, T. & ANDERSON, M. (1992). Human papillomavirus type 16 DNA in cervical smears as predictor of high-grade cervical cancer. *Lancet*, **339**, 959-960.

DYSON, N., HOWLEY, P.M., MUNGER, K. & HARLOW, E. (1989). The human papillomavirus 16 E7 oncoprotein is able to bind to the retinoblastoma gene product. *Science*, **243**, 934-937.

FOX, J.C., GRIFFITHS, P.D. & EMERY, V.C. (1992). Quantification of human cytomegalovirus DNA using the polymerase chain reaction. *J. Gen. Virol.*, (in press).

GILES, J.A., DEERY, A., CROW, J. & WALKER, P. (1989). The accuracy of repeat cytology in women with mildly dyskaryotic smears. *Br. J. Obstet. Gynaecol.*, **96**, 1067-1070.

KESSLER II (1976). Human cervical cancer as a venereal disease. *Cancer Res.*, **36**, 783-791.

SCHEFFNER, M., MUNGER, K., BYRNE, J.C. & HOWLEY, P.M. (1991). The state of the p53 and retinoblastima genes in human cervical carcinoma cell lines. *Proc. Natl. Acad. Sci. USA*, **88**, 5523-5527.

SCHEFFNER, M., WERNESS, B.A., HUIBREGSTE, J.M., LEVINE, A.J. & HOWLEY, P.M. (1991). The E6 oncoprotein encoded by human papillomavirus types 16 and 18 promotes the degradation of p53. *Cell*, **63**, 1129-1136.

SCHNEIDER, A., SAWADA, E., GISSMAN, L. & SHAH, K. (1987). Human papillomavirus in women with a history of abnormal Papanicolaou smears and their male partners. *Obstet. Gynecol.*, **69**, 554–562.

VAN DEN BRULE, A.J.C., WALBOOMERS, J.M.M., MAINE, M.D., KERNEMANS, P. & MEIJER, C.J.L.M. (1991). Difference in prevalence of human papillomavirus genotypes in cytomorphologically normal cervical smears is associated with a history of cervical intraepithelial neoplasia. *Int. J. Cancer*, **48**, 404–408.

ZUR HAUSEN, H. (1987). Papillomaviruses in human cancer. *Cancer*, **59**, 1692–1696.